# Optical Coherence Tomography Significance in Managing Early Onset of Optic Pathway Gliomas in Children Younger than 5 Years of Age—A Retrospective Study

**DOI:** 10.3390/children9091307

**Published:** 2022-08-28

**Authors:** Rossana Pavone, Carla Fonte, Iacopo Sardi, Roberto Caputo, Elisa Marziali, Fabio Mazzeo, Jacopo Secci, Alessia Bergamini, Salvatore De Masi, Maria Carmela Leo, Maria Luigia Censullo, Giacomo Maria Bacci

**Affiliations:** 1 Neuro-Oncology Unit, Department of Pediatric Oncology, Meyer Children’s Hospital, 50134 Florence, Italy; 2 Pediatric Ophthalmology Unit, Meyer Children’s Hospital, Viale Pieraccini 24, 50134 Florence, Italy; 3 Clinical Trial Center, Careggi Hospital, 50134 Florence, Italy; 4 Clinical Research and Study Design Office, Meyer Children’s Hospital, 50134 Florence, Italy

**Keywords:** optic pathway glioma, neurofibromatosis, optical coherence tomography, pRNFL, SD-OCT, OPG

## Abstract

We aimed to investigate the significance of optical coherence tomography (SD-OCT) in managing pediatric optic pathway gliomas (OPGs) in children younger than 5 years of age. A retrospective monocentric study was conducted. SD-OCT scans were obtained using the handheld iVue system to assess peripapillary retinal nerve fibre layer (pRNFL) thickness at three time points: baseline (OCT1), end of treatment (OCT2), and at last follow-up (OCT3). We compared the median value of pRNFL (and interquartile range—IQR) at different follow-up times and in different sub-groups (stable disease—SD, partial response—PR, and progression disease—PD). Thirteen children younger than 5 years of age were included. The Median follow-up time was 3.9 years (IQR 1.2). Six patients showed a pRNFL change of more than 10% during follow-up. Seven patients showed PD during follow-up. Median pRNFL at baseline was 81.5 µm (IQR 31.5); median pRNFL at the end of treatment was 73 µm (IQR 33); median pRNFL at last follow-up was 72 µm (IQR 38.5). The mean pRNFL at baseline was significantly lower than the mean normative values. Only subjects with PD showed pRNFL change close to statistical significance. This study confirms the role of SD-OCT in managing OPGs for therapeutic decisions and strategy planning of visual rehabilitation.

## 1. Introduction

Low-grade gliomas (LGGs) are low grade astrocytic tumours that can occur in the optic nerve, chiasm, optic tract, or optic radiations. They are commonly referred to as optic pathway gliomas (OPGs). Children with OPGs are frequently diagnosed after visual deficits are noted; other symptoms at presentation include proptosis or symptoms of the hypothalamic syndrome, correlating with the anatomic tumour location and side [1].

OPGs are classified by anatomic location (modified Dodge classification [2]) and by whether or not they are associated with neurofibromatosis type 1 (NF1). OPGs arise in 8–31% of individuals affected by NF1 [3,4], and significant differences have been shown in terms of tumour biology, outcome, prognostic factors, and neurocognitive deficits in this particular subgroup [5]. The optic nerve is also commonly affected in the NF1 population, whereas, in sporadic OPGs, chiasmal and retrochiasmal tumours are more frequently seen.

Multimodal treatment approaches for the management of OPGs in children have markedly contributed to improved survival [6]. Although both chemotherapy and radiotherapy can stabilise growth or even decrease the size of tumours, chemotherapy has fewer side effects than radiation therapy, especially in young patients.

The overall prognosis is excellent, with a high survival rate, even though some patients still experience progressive disease, significant morbidity, and visual loss despite chemo or radiotherapy [7].

Initial management of these lesions usually consists of close observation. Radiological (magnetic resonance imaging—MRI) and clinical (ophthalmological and endocrinological) follow-ups are needed, and first-line treatment is limited to patients exhibiting tumour growth at MRI or severe clinical symptoms.

Best corrected visual acuity (BCVA) and visual field loss from OPGs can occur despite improvement, stability, or progression of radiographic findings. 

The peripapillary retinal nerve fibre layer (pRNFL), the most proximal region of the afferent visual pathway, has been examined as a structural marker of visual integrity in patients with compressive optic neuropathy [8]. pRNFL thickness, as measured by spectral domain optical coherence tomography (SD-OCT), is closely correlated with low-contrast letter acuity in most children with OPGs and decreased pRNFL thickness may have abnormal BCVA, visual field loss, or both [9].

As most patients are younger than 5 years old at the time of diagnosis, accurate assessment of BCVA and visual fields can be challenging due to poor infant cooperation and child short attention spans. Limited compliance in this OPG patient population makes diagnosis and visual follow-up hard to define. SD-OCT is a noninvasive objective imaging technique of the retina and optic nerve head structure, offering a unique opportunity in this patient population: in fact, since the literature is relatively in agreement on the role of SD-OCT as a valid support for its role in defining the evolution of tumours involving optic pathways (OP), we sampled a population of pediatric OPGs to verify the actual support of pRNFL thickness assessment in children with early onset disease, OP involvement, and unreliable subjective data to monitor the evolution of the disease.

The aim of this study is, therefore, to explore SD-OCT significance in the early management of patients younger than 5 years of age affected by OPGs for clinical and rehabilitation purposes.

## 2. Materials and Methods

### 2.1. Patients

This retrospective analysis involved a pediatric OPG population aged 0–5 years recruited at Meyer’s Children University Hospital Neuro-Oncology Unit from 2014 to 2022. 

OPGs have been classified using the World Health Organization grading of central nervous system (CNS) tumours and in accordance with medical literature. Histological confirmation was not required in the case of OPGs clinical, radiological features evidence.

NF1 diagnosis was based on the criteria established by the National Institutes of Health Consensus Development Conference [10].

The main exclusion criteria were a history of any other ophthalmologic or neurologic disease that could affect visual function (such as cataracts, retinal detachment, retinopathy of prematurity, or glaucoma).

All patients underwent pRNFL thickness assessment before starting treatment (T0-OCT1), at the end of treatment (T1-OCT2), and at the last follow-up (T3-OCT3).

### 2.2. SD-OCT Procedure

The handheld iVue SD-OCT system (Optovue Inc., Fremont, CA, USA) has been used to determine and evaluate changes in pRNFL thickness. Since all our patients were noncompliant for in-office evaluation, we performed pRNFL thickness assessment during sedation while acquiring an MRI scan according to neuro-oncology protocols. After pupil dilation with Tropicamide 1% eye drops, the operator was positioned at the head of the bed, with the eyelid opened with a lid speculum, and the device was placed over the patient’s eye and positioned until optimal image quality was achieved. To optimise image quality, the horizontal and vertical B-scan images were displayed in real time and were adjusted to achieve optimal alignment. Once correct alignment and focus were achieved, a 6 mm × 6 mm rectangular scan centred on the optic nerve head was acquired using 1000 A-scan across 100 B-scan. Once image acquisition was complete, a horizontal B-scan and an en face volume intensity projection image were displayed to assess the quality and alignment of the image. pRNFL thickness measurements were automatically calculated with the built-in device iVue software v.3.0 (Optovue Inc., Fremont, CA, USA), providing a global average (M) and average thickness of two sectors: inferior (I) and superior (S). To better compare results across patients’ different tumour locations, only the pRNFL mean value was taken into consideration for progression analysis. According to the recent literature, a decrease of 10% [11] or more in pRNFL thickness between serial evaluations was considered indicative of relevant modification of the clinical evolution of the disease. A reference database for pRNFL normative values in children with Optovue technology was used to compare the pRNFL of our series to normal values [12].

### 2.3. Treatment Recommendations 

All patients underwent carboplatin–etoposide-based chemotherapy as initial treatment. Surgery was not mandatory for diagnosis confirmation, but it was sometimes performed for tumour debulking in case of OP compression.

### 2.4. Data Collection

We retrospectively collected the demographic information, tumour characteristics, cancer treatments (including surgical methods and chemotherapy regimen), ophthalmological history, as well as long-term follow-up data of patients with a diagnosis of sporadic or NF1-related OPGs.

The study protocol was submitted to the local Ethic Committee, and consent was obtained from parents/guardians according to national research ethics requirements.

### 2.5. Statistical Methods

This was a retrospective study conducted at Meyer Children Hospital in Florence from January 2014 to June 2022. The study was designed in accordance with the Helsinki Declaration. However, it was a pilot retrospective data-based study, and no formal sample size was calculated. 

For descriptive purposes, data are reported as the median and interquartile range (IQR) or as proportion. 

To analyse OCT values at different follow-up times, we compared the median value of the pRNFL (and IQR) at different follow-up times and in different subgroups (stable disease—SD, partial response—PR, and progression disease—PD). We also converted the difference of OCT values at different follow-up times into a significant difference (reduction of >10% compared with the basal value) and a not significant difference (reduction of ≤10% compared to the basal value). Therefore, we calculated the significant reduction in odds ratio (OR) (OCT3-OCT1 and OCT2-OCT1) in PR vs. SD and PD vs. SD patients, with 95% confidence intervals.

The comparisons were conducted using a nonparametric test. *p*-value less than and equal to 0.05 was considered statistically significant. The statistical analysis was carried out using the software Stata corp. vers. 17 (StataCorp LLC, College Station, TX, USA).

## 3. Results

A total of 13 children younger than 5 years of age at OPGs diagnosis were analysed: five males (38%), with median age, at diagnosis, of 2.4 years (Table 1). After a median follow-up of 3.9 years (IQR 1.2), one death (refractory Pilomyxoid astrocytoma, PD related) and seven PDs occurred. All PD occurred within 2 years from diagnosis (all *Pilomyxoid astrocytomas* and 3/4 *Pilocytic astrocytomas*).

OPGs diagnosis was defined only by radiological and clinical criteria in six cases (4/6 were NF1 patients). Histological OPGs confirmation before chemotherapy was performed in 7/13 (54%) children for the necessity of tumour debulking for OP compression. The histological diagnosis was Pilomyxoid astrocytoma in 3/7 (43%) patients and *Pilocytic astrocytoma* in 4/7 (57%).

An anatomical OPGs classification was proposed by Dodge et al. [13] in 1958, defining tumours as involving either the optic nerves alone (Stage 1), the chiasm (with or without nerve involvement) (Stage 2), and the hypothalamus or other adjacent structures (Stage 3). In 2008 Taylor et al. presented a modified Dodge classification (MDC) applying DC to MR scans and proposed an imaging-based method to allow a more detailed tumour description.

Tumour localizations according to modified Dodge classification [2] were H+ n = 10, 4 bL n = 1, and 2 bL n = 2. 

All patients underwent carboplatin–etoposide-based chemotherapy. Treatment was initiated for PD evidence on MRI, defined as tumour volume increase of >25% or clinical deterioration. None of them received radiotherapy.

Six out of thirteen patients showed a pRNFL change of more than 10% between the start and the end of treatment. Seven out of thirteen patients showed PD during follow-up. None of the four NF1 patients had PD during follow-up.

**Figure 1:**
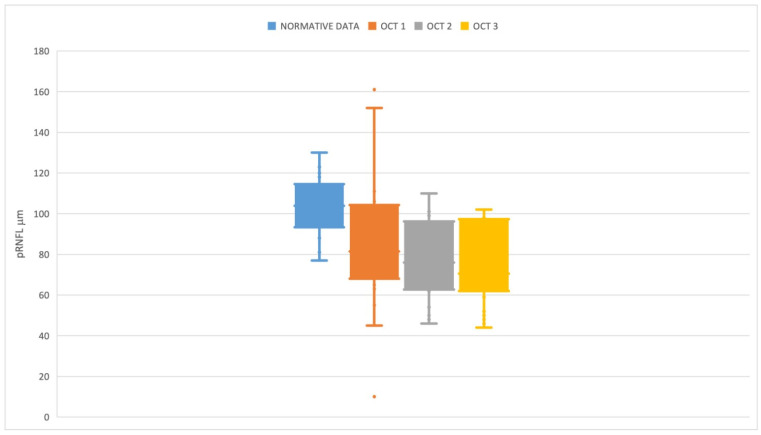
Box plot showing normative Optovue data (Blue plot) in relation to pRNFL at baseline (OCT1—Orange plot), at the end of treatment (OCT2—Grey plot), and at last follow-up (OCT3—Yellow plot).

Median pRNFL thickness at baseline (OCT1) was 81.5 µm (IQR 31.5); median pRNFL at the end of treatment (OCT2) was 73 µm (IQR 33); median pRNFL at last follow-up (OCT3) was 72 µm (IQR 38.5). As a general overview, the mean pRNFL at baseline for all patients with OPGs (NF1 and sporadic) was significantly lower than mean normative values (Figure 1), and a significant median decrease was noticed toward the end of treatment (*p* = 0.0017) and at the last follow-up (*p* = 0.0017) (Table 2).

The median differences in the pRNFL thickness between the two eyes were 1 or 2 μm at different follow-up intervals (OCT1, OCT2, and OCT3, respectively). These differences were observed for the total population and for the subjects with Chiasm–Hypothalamus localisation of cancer (the median difference between right eyes and left eyes was 2, 2, and 1 µm at first, second, and third measurement).

One patient with Bilateral Optic Tract localisation showed differences of 14, 1, and 2 μm, while one subject with Optic Nerves–Chiasm–Optic tracts localisation showed differences of 8, 9, and 13 µm.

Table 2 shows the median OCT and IQR values at baseline and at the different follow-up times (OCT2-OCT3), distributed by outcome (SD, PD, PR).

Only in PD patients the reduction in OCT values is close to statistical significance when calculated at the first and last follow-up (p = 0.0625 and p = 0.0625).

In PD vs. SD patients, the odds ratio (OR) to obtain a significant reduction in OCT3-OCT1 value was 12.0 (l.c. 95% 0.5–294.6), and the OR of the significant reduction in OCT2-OCT1 was 5.0 (l.c. 95% 0.3–91.5) (Table 3). The corresponding value in PR vs. SD patients was not estimable because of the small sample available.

## 4. Discussion

Several case series have demonstrated the variable clinical course of OPGs, and many recommendations for follow-up evaluations exist. Current modalities for identifying and following OPGs include clinical examination, visually evoked potentials (VEPs), and MRI.

Recently, Nuijts et al. [14] tried to define the accuracy and prognostic value of OCT in pediatric brain tumours, but the authors found scarce literature on this topic. Nevertheless, increasing evidence reported the importance of the pRNFL thickness analysis during the follow-up period of young patients affected by OPGs. Parrozzani et al. [15] have demonstrated that pRNFL analysis is superior to visual function assessment in 57 patients with NF1 and OPGs, but authors underline that a finding of a reduced pRNFL thickness should not be taken as definitive evidence of a clinically significant visual sensory dysfunction. The same author in [16], in 2018, reported the cutoff value of 76 μm, acquired with the Spectralis platform in awake children, as a marker for good visual acuity. Fisher et al. [6] evaluated visual outcomes after chemotherapy in NF1-associated OPGs and found that, although most subjects had some sort of visual improvement (32% of subjects) or stabilisation (40% of subjects) after chemotherapy and BCVA, they had worsened in 28% of subjects despite treatment. Fard et al. [17] in 2013 defined a loss of 8.62 µm from baseline as a marker of progression of optic pathway glioma, reinforcing the fact that considering a 10% decline in pRNFL thickness could be a viable option to monitor OPG evolution in our series.

In about 50% of prechiasmatic gliomas, there is a poor correlation between tumour growth and changes in BCVA. Therefore, monitoring pRNFL thickness may provide essential clinical information to modify or even defer treatment, especially in those children who cannot perform BCVA testing or perimetry. Avery et al. [9] tried to correlate abnormal BCVA (or visual field defect) and pRNFL thickness decrease in children with OPGs, demonstrating that most affected children with decreased pRNFL thickness had abnormal BCVA or visual field. The same author reported that some patients had normal BCVA despite a significantly decreased pRNFL thickness, possibly suggesting that BCVA or visual field loss may not have been detected with current techniques or the child had yet to manifest symptoms. On the other hand, if their vision testing were accurate, a normal pRNFL thickness showed a strong relation to normal BCVA and visual fields. These findings suggest that in children who are not cooperative enough to provide a reliable visual examination, a normal pRNFL thickness may be reassuring to the clinician that the patient likely has a normal BCVA and possibly visual field. In contrast, a reduced pRNFL thickness may or may not have a strong relation to clinical visual sensory function and thus should not be taken as absolute evidence of visual dysfunction. In recent years, Yanni et al. [18] and Bhoiwala et al. [12] published normative pediatric values for macular thickness, pRNFL thickness, and retinal thickness in healthy North American children using Spectralis and Optovue SD-OCT device, respectively: average global pRNFL thickness in children aged 5–15 years was 107.6 µm using Spectralis SD-OCT, while in patients of 5 years of age mean pRNFL was 103.93 ± 10.59 µm, which is significantly higher than normative data for healthy white adults [19]. According to the literature, the mean pRNFL thickness decreases approximately by 1.5 μm per decade in the healthy eyes of adults. Budenz et al. [20] estimated an average pRNFL thickness reduction of −0.20 µm/year, with average pRNFL thickness reduction ranging between −0.16 and −0.44 µm/year in most studies [21].

In this paper, we report the results of routine pRNFL evaluations in pediatric patients aged 0–5 years affected by OPGs treated using chemotherapy and with only OCT as a surrogate for visual function assessment. Since the sample size is limited for such a difficult aim, our data do not reach a statistical significance in all disease subgroups, but it is, however, possible to offer some considerations regarding our results:

Patients with PD present pRNFL changes that are more significant than in patients with PR and SD (Table 2), possibly indicating an actual role of SD-OCT serial evaluation in obtaining data on disease evolution also in this difficult population sample.

Although it is expected that pRNFL thickness can be lower than normative data for pediatric and adult populations be-cause of the natural course of the pathology affecting visual pathways, it is noteworthy that the baseline values are also well below these normative data (Figure 1). This finding may indicate that a degree of optic neuropathy is already pre-sent when the diagnosis is posed, even at an early age.

Another interesting consideration is that patients with stable disease also show a progressive pRNFL decrease during follow-up, but we cannot draw conclusions because of the lack of statistical significance. We can argue that, despite treatment, the axonal degeneration secondary to primitive tumour compression proceeds in parallel to the disease course and sets the basis for an additional, long-term optic neuropathy.

Nevertheless, this pilot study has important limitations: First, the limited sample number and the retrospective design do not allow us to draw conclusions, but we can report a tendency to provide more support to our aim. Second, there is a lack of a control group of patients with a brain tumour that did not require chemotherapy since there is evidence that axonal degeneration could continue well beyond the time when OPGs are believed to be symptomatic. The temporal relationship between pRNFL decline and visual loss or treatment toxicity has not been delineated by longitudinal studies; therefore, OCT results should not be used to make a clinical decision but may eventually serve as a reliable surrogate marker of vision and support.

## 5. Conclusions

Our paper shows a groups of very young patients with OPGs where the OCT was the only method to assess visual integrity because of a total lack of cooperation to subjective tests. We think that the main point of this paper resides in confirming the role of handheld OCT in managing the presentation and evolution of OPGs. Clinicians should be aware of this since anatomical support is the main pillar in starting the rehabilitation protocols as soon as the disease course allows it. Since brain plasticity can promote unpredictable results at a young age [22], it is of great importance to obtain data on anatomical support to vision in this particular subgroup of patients. If it is true that rehabilitation protocols are particularly challenging during the treatment period, it is likely that anatomical maintenance of pRNFL during the treatment and follow-up period can set the basis for reducing the amblyopic burden on the most affected eye. Although the role of OCT in PD is questionable for sporadic OPGs, its main role in response to treatment in NF1-related OPGs remains to define the anatomical support for VA and strategy planning for visual rehabilitation. Future larger prospective and longitudinal studies are warranted to confirm our preliminary results.

## Figures and Tables

**Table 1:** Patients’ characteristics. * NA—not available.

	Subgroups		Total
	NF1	Non-NF1	
**Number of Patients**	4	9	13
Females	4	4	8 (62%)
Males	-	5	5 (38%)
**Histology**	4	9	13
*Pilomyxoid astrocytoma*	-	3	3 (23%)
*Pilocytic astrocytoma*	-	4	4 (31%)
NA *	4	2	6 (46%)
**Modified Dodge Classification**			
2 bL	2	-	2 (15%)
4 bL	-	1	1 (8%)
H+	2	8	10 (77%)
**Events**	-	7	7
Progression	-	7	7
Second neoplasm	-	-	-
**Deaths**	-	1	1
Tumour	-	1	1
Other causes	-	-	-

**Table 2:** Median and IQR values of OCT at different follow-up times by disease status.

Variables	pRNFL (µm) Basal Median (IQR)	pRNFL (µm) First Follow-Up Median (IQR)	pRNFL (µm) Last Follow-Up Median (IQR)	*p* (Last Follow-Up Basal)	*p* (First Follow-Up Basal)
Progression Disease (PD)	80.5 (20)	66 (31)	63.5 (17)	0.0625	0.0625
Stable Disease (SD)	103.5 (66.5)	95 (42)	98 (42.5)	0.1250	0.1250
Partial Response (PR)	104 (32.5)	96.5 (30.5)	95.5 (29)	0.1250	0.1250
Total	81.5 (31.5)	73 (33)	72 (38.5)	0.0017	0.0017

**Table 3:** OR of significant reduction in pRNFL (>10%) by disease status.

	OCT3-OCT1			OCT2-OCT1		
	N		OR Difference > 10% (l.c. 95%)	N		OR Difference > 10% (l.c. 95%)
Variable	>10%	<=10%		>10%	<=10%	
SD	1	2	1.0	1	2	1.0
PD	6	1	12.0 (0.5–294.6)	5	2	5.0 (0.3–91.5)
PR	0	3	1.5^−7^ (not estimable)	0	3	4.2^−8^ (not estimable)
Tot	7	6		6	7	

## Data Availability

Not applicable.

## References

[1] Binning M.J., Liu J.K., Kestle J.R., Brockmeyer D.L., Walker M.L. (2007). Optic pathway gliomas: A review. Neurosurg. Focus.

[2] Taylor T., Jaspan T., Milano G., Gregson R., Parker T., Ritzmann T., Benson C., Walker D. (2008). Radiological classification of optic pathway gliomas: Experience of a modified functional classification system. Br. J. Radiol..

[3] Cummings T.J., Provenzale J.M., Hunter S.B., Friedman A.H., Klintworth G.K., Bigner S.H., McLendon R.E. (2000). Gliomas of the optic nerve: Histological, immunohistochemical (MIB-1 and p53), and MRI analysis. Acta Neuropathol..

[4] Czyzyk E., Jozwiak S., Roszkowski M., Schwartz R.A. (2003). Optic pathway gliomas in children with and without neurofibromatosis 1. J. Child Neurol..

[5] Listernick R., Ferner R.E., Liu G.T., Gutmann D.H. (2007). Optic pathway gliomas in neurofibromatosis-1: Controversies and recommendations. Ann. Neurol..

[6] Fisher M.J., Loguidice M., Gutmann D., Listernick R., Ferner R., Ullrich N.J., Packer R.J., Tabori U., Hoffman R.O., Ardern-Holmes S.L. (2012). Visual outcomes in children with neurofibromatosis type 1-associated optic pathway glioma following chemotherapy: A multicenter retrospective analysis. Neuro-Oncol..

[7] Via P.D., Opocher E., Pinello M.L., Calderone M., Viscardi E., Clementi M., Battistella P.A., Laverda A.M., Da Dalt L., Perilongo G. (2007). Visual outcome of a cohort of children with neurofibromatosis type 1 and optic pathway glioma followed by a pediatric neuro-oncology program. Neuro-Oncol..

[8] Avery R.A., Rajjoub R.D., Trimboli-Heidler C., Waldman A.T. (2015). Applications of optical coherence tomography in pediatric clinical neuroscience. Neuropediatrics.

[9] Avery R.A., Hwang E.I., Ishikawa H., Acosta M.T., Hutcheson K.A., Santos D., Zand D.J., Kilburn L.B., Rosenbaum K.N., Rood B.R. (2014). Handheld optical coherence tomography during sedation in young children with optic pathway gliomas. JAMA Ophthalmol..

[10] Legius E., Messiaen L., Wolkenstein P., Pancza P., Avery R.A., Berman Y., Blakeley J., Babovic-Vuksanovic D., Cunha K.S., Ferner R. (2021). Revised diagnostic criteria for neurofibromatosis type 1 and Legius syndrome: An international consensus recommendation. Genet. Med. Off. J. Am. Coll. Med. Genet..

[11] Avery R.A., Cnaan A., Schuman J.S., Trimboli-Heidler C., Chen C.-L., Packer R.J., Ishikawa H. (2015). Longitudinal Change of Circumpapillary Retinal Nerve Fiber Layer Thickness in Children With Optic Pathway Gliomas. Am. J. Ophthalmol..

[12] Bhoiwala D.L., Simon J.W., Raghu P., Krishnamoorthy M., Todani A., Gandham S.B., Simmons S. (2015). Optic nerve morphology in normal children. J. Am. Assoc. Pediatric Ophthalmol. Strabismus.

[13] Dodge H.W., Love J.G., Craig W.M., Dockerty M.B., Kearns T.P., Holman C.B., Hayles A.B. (1958). Gliomas of the optic nerves. AMA Arch. Neurol. Psychiatry.

[14] Nuijts M.A., Imhof S.M., Veldhuis N., Dekkers C.C., Schouten-van A.Y.N.M., Stegeman I. (2021). The diagnostic accuracy and prognostic value of OCT for the evaluation of the visual function in children with a brain tumour: A systematic review. PLoS ONE.

[15] Parrozzani R., Clementi M., Kotsafti O., Miglionico G., Trevisson E., Orlando G., Pilotto E., Midena E. (2013). Optical coherence tomography in the diagnosis of optic pathway gliomas. Investig. Ophthalmol. Vis. Sci..

[16] Parrozzani R., Miglionico G., Leonardi F., Pulze S., Trevisson E., Clementi M., Opocher E., Licata V., Viscardi E., Pilotto E. (2018). Correlation of peripapillary retinal nerve fibre layer thickness with visual acuity in paediatric patients affected by optic pathway glioma. Acta Ophthalmol..

[17] Fard M.A., Fakhree S., Eshraghi B. (2013). Correlation of optical coherence tomography parameters with clinical and radiological progression in patients with symptomatic optic pathway gliomas. Graefe’s Arch. Clin. Exp. Ophthalmol..

[18] Yanni S.E., Wang J., Cheng C.S., Locke K.I., Wen Y., Birch D., Birch E.E. (2013). Normative reference ranges for the retinal nerve fiber layer, macula, and retinal layer thicknesses in children. Am. J. Ophthalmol..

[19] Meyer J., Karri R., Danesh-Meyer H., Drummond K., Symons A. (2021). A normative database of A-scan data using the Heidelberg Spectralis Spectral Domain Optical Coherence Tomography machine. PLoS ONE.

[20] Budenz D.L., Anderson D.R., Varma R., Schuman J., Cantor L., Savell J., Greenfield D.S., Patella V.M., Quigley H.A., Tielsch J. (2007). Determinants of normal retinal nerve fiber layer thickness measured by Stratus OCT. Ophthalmology.

[21] Leung C.K., Yu M., Weinreb R.N., Ye C., Liu S., Lai G., Lam D.S. (2012). Retinal nerve fiber layer imaging with spectral-domain optical coherence tomography: A prospective analysis of age-related loss. Ophthalmology.

[22] Fazzi E., Micheletti S., Calza S., Merabet L., Rossi A., Galli J., Accorsi P., Alessandrini A., Bertoletti A., Campostrini E. (2021). Early visual training and environmental adaptation for infants with visual impairment. Dev. Med. Child Neurol..

